# Cerebral structural alterations in the patients undergoing postherpetic neuralgia: A VBM–MRI study

**DOI:** 10.1002/ibra.12027

**Published:** 2022-03-29

**Authors:** Li Niu, Yi Hu, Cheng‐Dong Yuan, Xing‐Yan Wu, Lei‐Lei Zheng, Yi Zhang

**Affiliations:** ^1^ Department of Anesthesiology The Second Affiliated Hospital of Zunyi Medical University Zunyi Guizhou China; ^2^ Key Laboratory of Anesthesia and Organ Protecting of Guizhou Province Zunyi Guizhou China; ^3^ Department of Anesthesiology Zunyi Medical University Zunyi Guizhou China

**Keywords:** brain, gray matter, magnetic resonance imaging, postherpetic neuralgia, voxel‐based morphometry

## Abstract

This study aimed to investigate the changes in gray matter (GM) volume and density in patients with postherpetic neuralgia (PHN). Using voxel‐based morphometry (VBM), the differences in cerebral GM volume and concentration between 25 PHN patients and 25 healthy controls with similar gender ratios, ages, and education were compared. Meanwhile, correlation analysis was performed between the value of GM volume/concentration in the brain areas with discrepancy and the visual analog scale (VAS) score/lesion duration. The global GM volume in PHN patients was lower than that of healthy controls, while the total volume of cerebrospinal fluid in PHN patients was higher than that of healthy controls. In PHN patients, the GM volume decreased in the striatum, cerebellum, precentral gyrus, middle frontal gyrus, parahippocampal gyrus, postcentral gyrus, and so forth; the GM concentration decreased in the striatum, insula, middle and posterior cingulate, and superior temporal gyrus. There was a negative correlation between GM concentration in the right parahippocampal gyrus and the VAS in patients with PHN. In PHN patients, GM volume and density in the brain regions involved in nociceptive sensation, pain perception, and integration decreased significantly. The interaction between chronic pain of PHN and alteration of the cerebral structure may contribute to the occurrence and development of PHN.

## Graphical abstract

Using voxel‐based morphometry, we found that gray matter volume and density were significantly reduced in several brain regions, including those directly involved in pain. This interaction between brain structure and postherpetic neuralgia (PHN) chronic pain may be one of the important mechanisms for the occurrence and development of PHN.

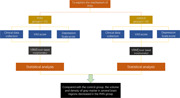

## 1 INTRODUCTION

Postherpetic neuralgia (PHN) is persistent intractable pain in the affected area for more than one month after recovery from herpes zoster.^1^ Its clinical manifestations include persistent or intermittent burning pain at the original lesion and stabbing or sharp shooting pain with hyperalgesia or allodynia.^2^ PHN is one of the most common complications of herpes zoster. Persistent severe pain has a huge impact on a patient's physical function, mental health, and social communication ability, and also places a heavy burden on patients' families and on society.

To date, there is no effective treatment for PHN because of its unclear pathogenesis. Our previous study, using functional magnetic resonance imaging (fMRI), showed that PHN could significantly affect the function of multiple brain areas in patients, which proved that the central mechanism is involved in the long‐term maintenance of PHN.^3^ Whether chronic pain caused by PHN can change the brain structures of patients remains to be further studied. In recent years, with the development of neuroimaging, magnetic resonance imaging (MRI) technology has provided powerful technical support for exploring the central changes caused by chronic pain. At present, voxel‐based morphometry (VBM) is mainly used to detect the structural changes of white matter and gray matter (GM) in the central nervous system.^4^ Therefore, in the present study, the changes in GM volume and density in the whole brain of PHN patients were observed using VBM, and the mechanism underlying long‐term maintenance and development of PHN is further explored from the perspective of cerebral structure alterations.

## 2 METHODS

### 2.1 Participants

This study was approved by the medical ethics committee of the Affiliated Hospital of Zunyi Medical University. All the participants were informed about the topic of this study; they participated voluntarily and signed the informed consent. Postherpetic neuralgia group (PHN group): from November 2014 to June 2015, 25 patients with PHN were selected from the Department of pain, the Affiliated Hospital of Zunyi Medical College, Guizhou Province. The inclusion criteria were as follows: (1) younger than 80 years of age; (2) PHN diagnosed independently by two senior pain physicians according to the International Association for the study of Pain (IASP) criteria; disease course of more than 2 months, visual analog scale (VAS) score higher than 5, and lesion area on the left side of the body; (3) no history of severe cardiovascular and cerebrovascular diseases, and no history of the nervous system and mental illnesses; (4) right‐handed; (5) no obvious cerebral structural abnormality found on MRI scan; (6) Beck Depression Inventory (BDI) score lower than 14; and (7) no contraindications for MRI scan. The exclusion criteria were as follows: patients with a history of epilepsy, craniocerebral trauma, identification of cerebral infarction on MRI plain scan, presence of a large number of demyelinating lesions, and other obvious structural lesions. The patients were asked to stop using the drugs 12 h before scanning.

The inclusion criteria of 25 healthy controls were as follows: (1) sex, age composition, and education level matched with the PHN group; (2) no history of chronic pain, neuropsychiatric disorders, or severe cardiovascular and cerebrovascular diseases; (3) right‐handed; (4) Beck Depression Inventory < 14; and (5) no obvious brain structural abnormalities on MRI scan.

### 2.2 Clinical evaluation

Collection of general clinical data, and determination of the VAS score and the Depression Scale score were performed before MRI scanning. MRI scanning was performed in the imaging center of Affiliated Hospital of Zunyi Medical University, Guizhou Province. The data processing and analysis were completed under the guidance of technicians from the imaging center of Affiliated Hospital of Zunyi Medical College.

### 2.3 Magnetic resonance data acquisition

A Ge signal HDxt 3.0 T magnetic resonance imaging system (Erlangen) with eight‐channel phased‐array coils were used for MRI scanning. The scanning sequence included axial T1‐weighted water suppression images (T1‐flair), axial T2‐weighted water suppression images (T2‐FLAIR), 3D T1‐weighted structural images acquired by 3D Bravo, and whole‐brain sagittal imaging.

### 2.4 Data postprocessing

VBM data were processed on the platform of MATLAB 2018a using the VBM8 tool in the spm8 software package. The 3D T1‐weighted images of all participants were divided into images of GM, white matter, and cerebrospinal fluid (CSF). The images were registered through differential‐algebraic neural registration using exponential Lie algebra, the Dartel tool for spatial standardization, and Gaussian smoothing. 3D T1WI data were imported into VBM8 software for parameter calculation to obtain the volume density data of white matter, GM, and CSF of each participant. The brain regions of the two groups with different GM volumes and densities were compared and marked in the coordinate space of the Montreal Neurological Institute (MNI).

### 2.5 Statistical analysis

General data were analyzed using SPSS19.0 software, and measurement data were expressed as mean ± standard deviation. Count data were compared using the *χ* ^2^ test; *p* < 0.05 was considered statistically significant. The S‐w method was used for the normality test, and an independent‐sample *t* test was used for comparison of the mean values between samples with a normal distribution. *p* < 0.05 was considered statistically significant. The comparison of MRI data groups was performed using a two‐sample *t* test, *p* < 0.001 (uncorrected), and the difference was considered statistically significant in the set with a voxel above 50. The brain area with significant differences in GM volume between the two groups was analyzed by Pearson's correlation analysis on the basis of the VAS score and disease course, and *p* < 0.05 was considered statistically significant.

## 3 RESULTS

### 3.1 Comparison of demographic and clinical data between the two groups

There was no significant difference in age, sex ratio, and education level between the PHN group and the control group (*p* > 0.05), but the BDI score of the PHN group was higher than that of the healthy control group (*p* < 0.05). The comparison of clinical data of the two groups is shown in Table 1.

**Table 1:** Comparison of clinical data between PHN patients and healthy controls, $\bar{x}\pm s,n=25$

	PHN group	Healthy controls	*T* or $\chi^{2}$	*p* Value
Age (year)	63.0 ± 7.51	61.68 ± 8.09	0.598	0.553
Sex ratio (male/female)	13/12	10/15	0.725	0.571
Education level (year)	4.88 ± 2.18	5.72 ± 2.01	−1.414	0.164
VAS score	6.53 ± 1.46	—		—
BDI score	6.36 ± 2.36	2.44 ± 1.42		<0.001
Course of disease (month)	5.12 ± 6.17	—		—

Abbreviations: BDI, Beck Depression Inventory; PHN, postherpetic neuralgia; VAS, visual analog scale.

### 3.2 GM, white matter, CSF, and whole‐brain volume

The total volume of GM was significantly reduced (*p* = 0.007) and the CSF was significantly increased (*p* < 0.001) in the PHN groups. There was no significant difference in the total volume of white matter and the total volume of the whole brain between the two groups (Table 2).

**Table 2:** Comparison of global gray matter volume, white matter volume, and cerebrospinal fluid total volume, $\bar{x}\pm s,n=25$

	PHN group	Healthy controls	*T* value	*p* Value
Total gray matter volume	512.88 ± 53.1	565.34 ± 53.51	−2.87	0.007
Total white matter volume	589.18 ± 59.5	580.16 ± 46.75	0.491	0.627
Total cerebrospinal fluid	246.54 ± 29.29	206.81 ± 28.84	3.985	<0.001
Whole‐brain volume	1348.59 ± 123	1352.31 ± 110.94	−0.093	0.927

### 3.3 The volume and density of GM

**Figure 1:**
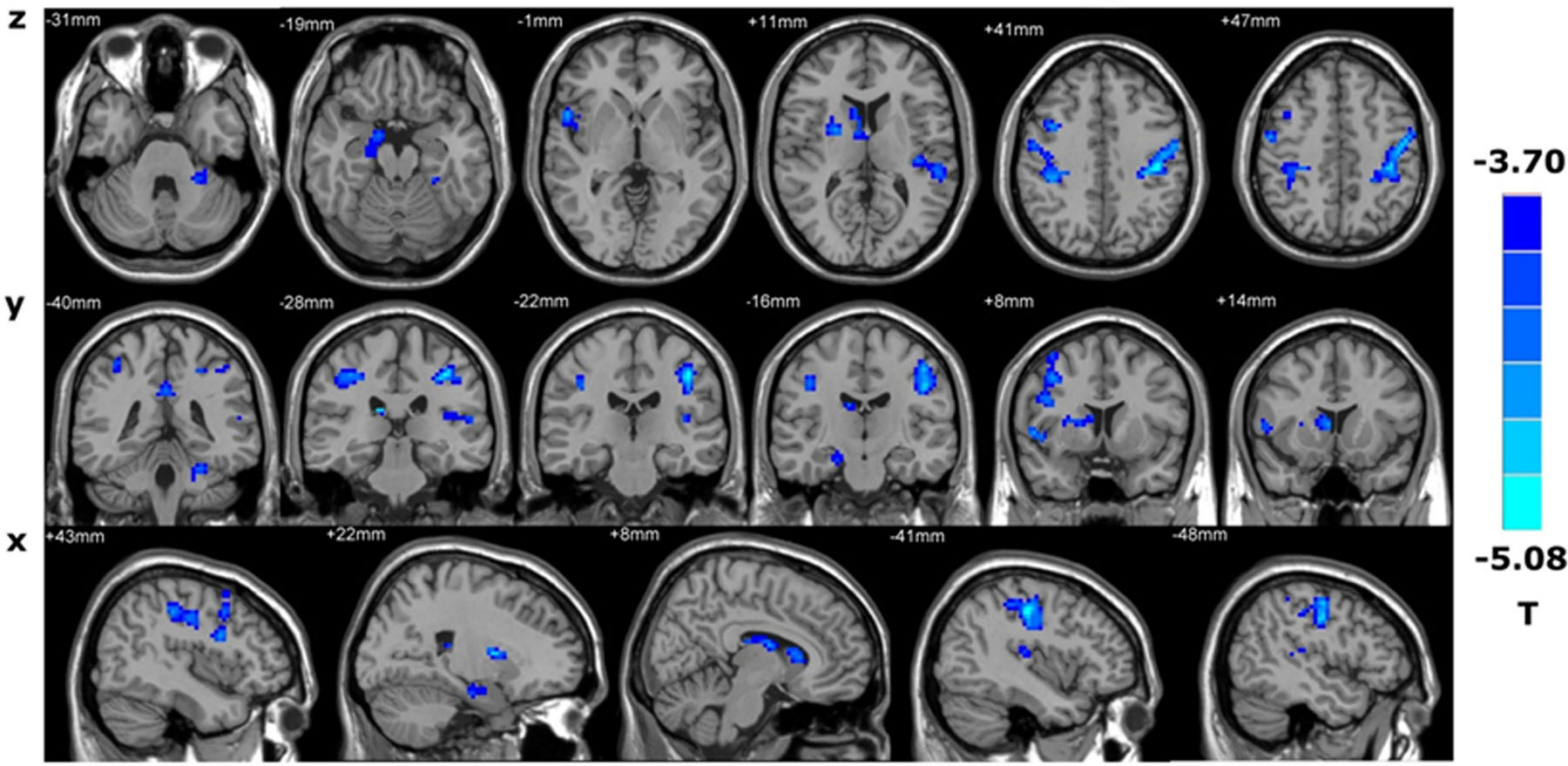
Brain regions with decreased gray matter volume in PHN patients compared with controls. PHN: postherpetic neuralgia [Color figure can be viewed at wileyonlinelibrary.com]

**Figure 2:**
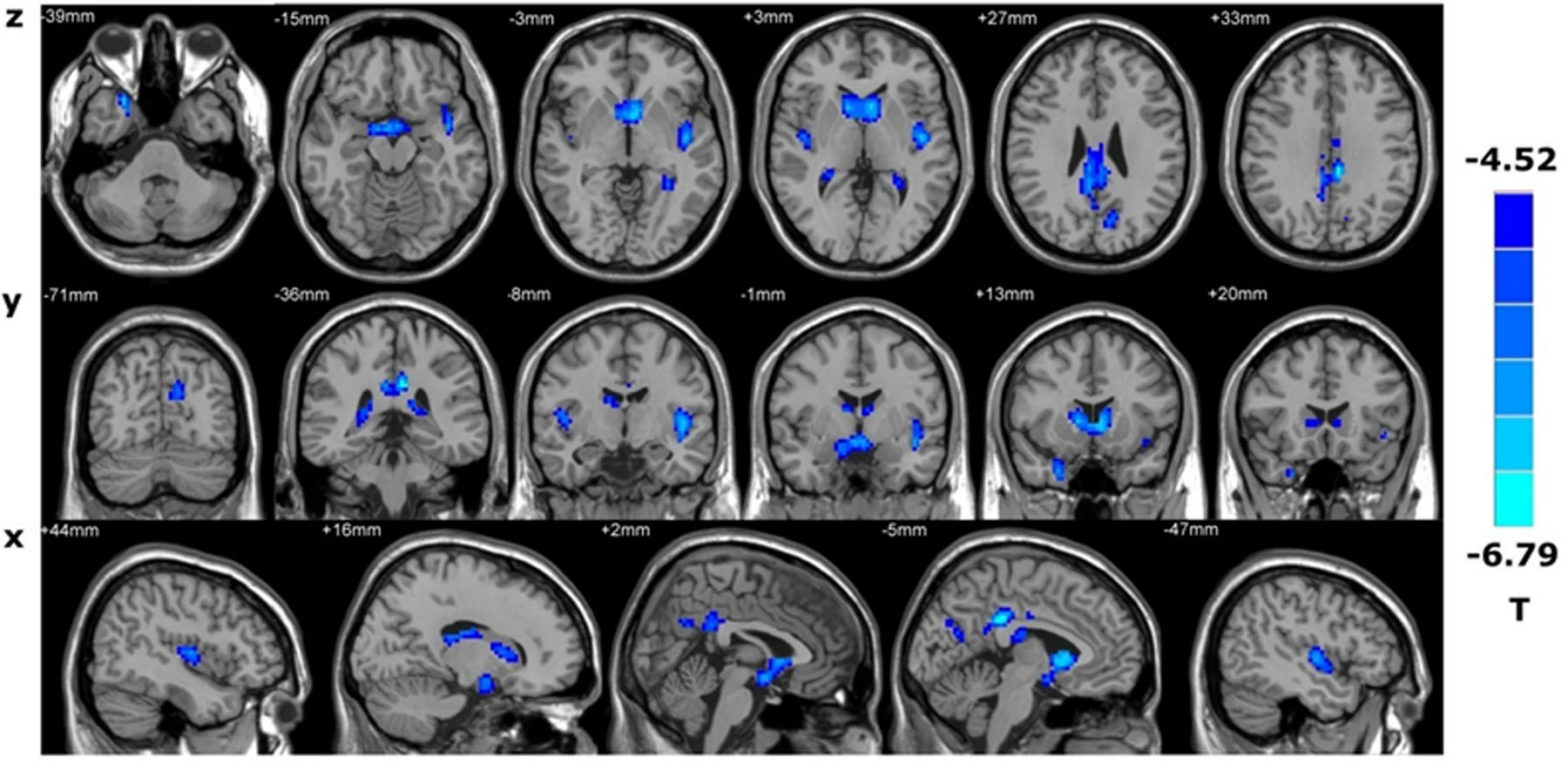
Brain regions with decreased gray matter concentration in PHN patients compared with controls. PHN, postherpetic neuralgia [Color figure can be viewed at wileyonlinelibrary.com]

The GM volume of PHN patients decreased significantly in the bilateral posterior central gyrus, the right anterior central gyrus, the right parahippocampal gyrus, the right caudate nucleus, the putamen, the right middle frontal gyrus, the left anterior cerebellar lobe, the left middle cingulate gyrus, and the left superior temporal gyrus (*p* < 0.001, uncorrected, cluster size > 50), as shown in Figure 1 and Table 3. The GM density in the bilateral caudate nucleus, the bilateral posterior cingulate gyrus, the bilateral insular lobe, the left cuneiform lobe,  the left superior temporal gyrus, and the left middle cingulate gyrus was significantly lower in PHN patients (*p* < 0.001, uncorrected, cluster size > 50) (Figure 2, Table 4).

**Table 3:** Distribution of brain regions with decreased GMV in PHN patients compared with controls

	MNI coordinates				
Brain area	*x*	*y*	*z*	Voxel	*T* value
Left anterior lobe of the cerebellum	−27	−36	−27	37	−4.34
Right parahippocampal gyrus	15	−3	−18	26	−4.24
Right putamen	25	4	10	35	−4.2
Right caudate nucleus	10	17	5	43	−4.23
Left superior temporal gyrus	−39	−24	12	36	−4.22
Right middle frontal gyrus	39	10	42	54	−4.49
Right anterior central gyrus	54	−6	45	71	−4.6
Middle cingulate gyrus	0	−39	33	22	−4.25
Left central posterior gyrus	−39	−24	42	235	−5.082
Right central posterior gyrus	40	−29	42	102	−4.56

Abbreviations: GMV, gray matter volume; MNI, Montreal Neurological Institute; PHN, postherpetic neuralgia.

**Table 4:** Distribution of brain regions with decreased GM concentration in PHN patients compared with controls

		MNI coordinates				
No.	Brain area	*x*	*y*	*z*	Voxel	*T* value
1	Left caudate nucleus	−5	9	1	106	−6.27
2	Right caudate nucleus	8	9	3	102	−5.65
3	Left posterior cingulate gyrus	−6	−36	30	38	−6.79
4	Right posterior cingulate gyrus	5	−42	30	46	−5.84
5	Left middle cingulate gyrus	−6	−34	36	36	−5.99
6	Left superior temporal gyrus	−43	−13	−6	71	−5.47
7	Left cuneiform lobe	−15	−63	21	46	−5.69
8	Left insular lobe	−45	−9	3	53	−6.29
9	Right insular lobe	45	−12	6	79	−5.796

Abbreviations: GM, gray matter; MNI, Montreal Neurological Institute; PHN, postherpetic neuralgia.

### 3.4 Correlation analysis of GM volume/density in the PHN group and clinical variables

**Figure 3:**
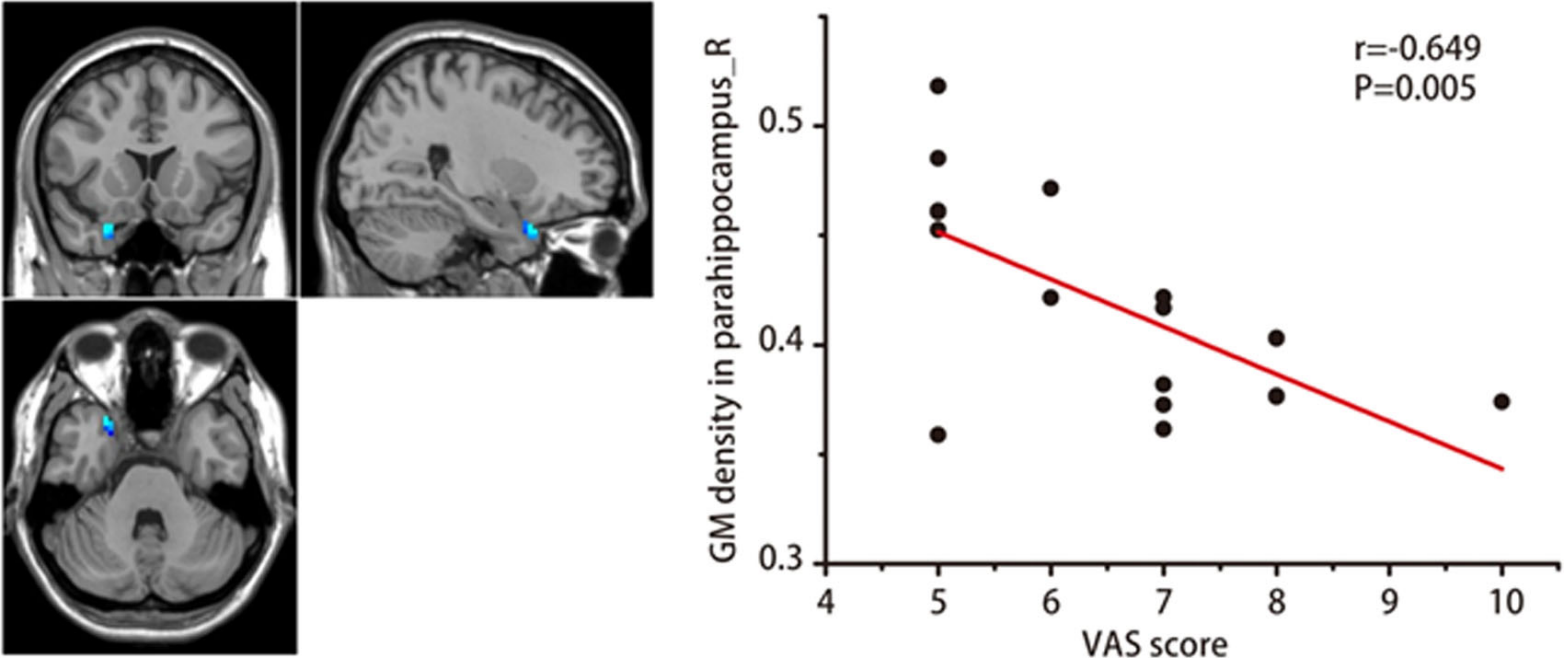
The GM density in the right parahippocampal gyrus is negatively correlated with the VAS score of PHN patients. GM: gray matter; PHN: postherpetic neuralgia; VAS, visual analog scale [Color figure can be viewed at wileyonlinelibrary.com]

Correlation analysis showed that there was a significant negative correlation between GM density in the right parahippocampal gyrus and the VAS score in the PHN group (*r* = −0.649, *p* = 0.005), as shown in Figure 3.

## 4 DISCUSSION

Compared with the traditional VBM algorithm, the DARTEL algorithm formulated by Ashburner et al. can make up for the defects of traditional VBM technology in image registration. Therefore, it is more sensitive to changes in cerebral density and volume and thus can more accurately display the brain structure.^5^ Studies have shown that compared with white matter, GM in the brain is more susceptible to long‐term chronic pain, such as trigeminal neuralgia and chronic low back pain, which can lead to a decrease in GM volume.^6^, ^7^ Therefore, our study used VBM‐Dartel to detect changes in the central nervous system in PHN patients and found significant changes in GM volume and density in multiple regions associated with pain.

The transformation of peripheral nociception into pain perception requires multiple levels of integration. First, it is conducted along the spinothalamic tract to the multiple nuclei in the posterior thalamus and then projects to the medial parietal insula (including the somatosensory cortex), the middle cingulate gyrus, and the posterior insula.^8^ Hence, these brain areas are the predominant areas that respond to noxious stimuli. Among them, the posterior insula is one of the regions most closely related to pain. Electrical stimulation of the posterior insula can cause acute pain,^9^ and chronic injury in the posterior insula can lead to neuropathic pain.^10^ Also, several VBM studies have shown that the GM of the insular lobe of patients with chronic neuropathic pain is significantly reduced, including trigeminal neuralgia,^11^ chronic low back pain,^6^ and migraines.^12^ These damages may be related to the long‐term maintenance of pain after the recovery of the skin lesions induced by herpes zoster. The primary sensory cortex (S1 area) is located in the central posterior gyrus of the apical insular area; PHN pain can directly activate this area.^13^ Studies also show that the S1 area of ​​patients with chronic pain shows significant changes in GM,^14^ which may be mainly related to PHN paresthesia and hyperalgesia. The anterior cingulate gyrus, the middle cingulate gyrus, and the posterior cingulate gyrus participate in the downward pain regulation network of the GM around the midbrain aqueduct,^15^ which can be activated by various types of pain, and play a very crucial role in the integration process of pain and emotional coding.^16^ After 11 days of repeated pain stimulation of healthy volunteers, the GM density of the anterior cingulate gyrus significantly reduced.^17^ Meanwhile, under pathological conditions such as irritable bowel syndrome,^18^ chronic pain after herpes simplex virus infection,^19^ mania,^20^ and other pathological conditions, GM volume reduction may also occur in the anterior cingulate gyrus and the middle cingulate gyrus. The structural disorder of the cingulate gyrus area is considered to be one of the key factors of depression caused by chronic pain,^21^ and therefore determines the clinical manifestations of depression in PHN patients to a certain extent.

In some unconscious states, although painful stimuli can activate the aforementioned brain regions, these sensory responses cannot be translate into pain perception.^22^ The formation of truly pain perception also requires the second level of nociceptive sensation integration. The brain areas involved in secondary integration include the anterior insula, the prefrontal lobe, the posterior parietal lobe, the striatum, the hippocampus, and the parahippocampal gyrus, the cerebellum, and the temporal–parietal junction area.^23^ These brain areas are not the direct projection regions of the spinothalamic tract; thus, direct stimulation in these areas will not induce pain and selective lesions in these areas have no obvious analgesic effect. Our study found a remarkable decrease in the GM volume/density in the brain regions associated with secondary integration in PHN patients. More specifically, the GM density of the right parahippocampal gyrus was also negatively correlated with the pain intensity of PHN patients. Therefore, It is implicated that chronic pain of PHN significantly affects these secondary integrated brain regions, which is consistent with the previous results of most VBM studies of other chronic neuropathic pain.^24^

The role of these changes in the structure of secondary integrated brain regions in PHN and other neuropathic pain is still unclear: Although most of the current academic consensus supports the idea that chronic pain causes changes in brain fitness, other studies have found that damage to certain brain regions can also contribute to chronic pain, and in most cases, the relationship between the two may be mutually reinforcing. For example, axons from the insula are connected to the frontal cortex and participate in the integration process of sensory information from back to front, which is one of the components of the attention network and the salience network.^25^ In addition, it is still inconclusive as to how structural changes may affect the maintenance and development of PHN, because it is impossible to reverse the structural abnormalities of brain regions through specific interventions. Therefore, at this stage, we can only speculate based on the functions of the corresponding brain regions, which can be further verified by animal models in future studies.

In summary, the volume and density of GM decreased significantly in the brain regions involved in pain perception, perception and integration in PHN patients, including cingulate gyrus, posterior central gyrus and the participating insula, frontal lobe, superior temporal gyrus, striatum and cerebellum. The interaction between brain structure and PHN chronic pain may be one of the important mechanisms underlying the occurrence and development of PHN.

## AUTHOR CONTRIBUTIONS

Niu Li contributed to the interpretation and writing of the study. Zhang Yi contributed to the implementation of the study. Yuan Chengdong contributed to the modification of the format of the article. Xingyan Wu contributed to the data analysis. All authors participated in drafting and revising the article, ultimately approving the upcoming edition and agreeing to be responsible for all aspects of the work.

## CONFLICTS OF INTEREST

The authors declare no conflicts of interest.

## ETHICS STATEMENT

The experiments involving the subjects were approved by the Medical Ethics Committee of the Affiliated Hospital of Zunyi Medical University and carried out after the Declaration of Helsinki (approval no. KLL‐2019‐035).

## Data Availability

The data that support the findings of this study are available from the corresponding author upon reasonable request.
